# Bifidobacterial Distribution Across Italian Cheeses Produced from Raw Milk

**DOI:** 10.3390/microorganisms7120599

**Published:** 2019-11-21

**Authors:** Christian Milani, Giulia Alessandri, Leonardo Mancabelli, Gabriele Andrea Lugli, Giulia Longhi, Rosaria Anzalone, Alice Viappiani, Sabrina Duranti, Francesca Turroni, Maria Cristina Ossiprandi, Douwe van Sinderen, Marco Ventura

**Affiliations:** 1 Laboratory of Probiogenomics, Department of Chemistry, Life Sciences, and Environmental Sustainability, University of Parma, 43124 Parma, Italy; christian.milani@unipr.it (C.M.); leonardo.mancabelli@genprobio.com (L.M.); gabrieleandrea.lugli@unipr.it (G.A.L.); sabrina.duranti@unipr.it (S.D.); francesca.turroni@unipr.it (F.T.); 2 Department of Veterinary Medical Science, University of Parma, 43124 Parma, Italy; giulia.alessandri1@studenti.unipr.it (G.A.); mariacristina.ossiprandi@unipr.it (M.C.O.); 3 GenProbio srl, 43124 Parma, Italy; giulia.longhi@studenti.unipr.it (G.L.); rosaria.anzalone@genprobio.com (R.A.); alice.viappiani@genprobio.com (A.V.); 4 Microbiome Research Hub, University of Parma, 43124 Parma, Italy; 5 APC Microbiome Institute and School of Microbiology, Bioscience Institute, National University of Ireland, T12 YT20 Cork, Ireland; d.vansinderen@ucc.ie

**Keywords:** bifidobacteria, metagenomics, profiling, microbiota, cheese

## Abstract

Cheese microbiota is of high industrial relevance due to its crucial role in defining the organoleptic features of the final product. Nevertheless, the composition of and possible microbe–microbe interactions between these bacterial populations have never been assessed down to the species-level. For this reason, 16S rRNA gene microbial profiling combined with internally transcribed spacer (ITS)-mediated bifidobacterial profiling analyses of various cheeses produced with raw milk were performed in order to achieve an in-depth view of the bifidobacterial populations present in these microbially fermented food matrices. Moreover, statistical elaboration of the data collected in this study revealed the existence of community state types characterized by the dominance of specific microbial genera that appear to shape the overall cheese microbiota through an interactive network responsible for species-specific modulatory effects on the bifidobacterial population.

## 1. Introduction

Metagenomic analyses have only recently been applied to study bacterial populations harbored by cheese. Most metagenomic investigations rely on 16S rRNA gene-based microbial profiling due to its lower costs and accurate taxonomic assignment down to the genus level. Moreover, metagenomics does not require bacterial cultivation, thus allowing retrieval of complete taxonomic profiles, including bacteria that currently cannot be cultivated. This methodology has been applied to a range of cheeses, such as Tomme d’Orchies [1], mozzarella [2], Mexican cheeses [3], oscypek (Polish cheese) [4], Croatian cheeses [5], Belgian cheeses [6], Pico cheese (artisanal Azorean food) [7], caciocavallo [8], plaisentif [9] and Italian grana-like cheese [10]. Nevertheless, none of these studies reported data at sub-genus taxonomic levels, because the taxonomic depth achieved with 16S rRNA gene sequencing is limited in this regard. To overcome this limitation, a cost-effective tool based on sequencing of the internally transcribed spacer (ITS) region for accurate subspecies level profiling of the bifidobacterial population was recently described [11].

Members of the genus *Bifidobacterium* have been shown to represent common gut colonizers of many occupants on the mammalian branch of the tree of life [12]. During the last two decades bifidobacteria have been extensively studied for their contribution to elicit a range of host health benefits, specifically during the first stages of life [13,14,15]. Among such reported health-promoting activities, bifidobacteria have been associated with key physiological aspects in infants, for example, the induction of mucus layer production and development of the gastro-intestinal tract, along with protection against (opportunistic) pathogens and maturation of the immune system [16,17,18]. However, positive biological roles have also been reported in adults, where their involvement in the breakdown of indigestible food components through expansion of the gut glycobiome is also considered of crucial relevance [19,20,21]. For these reasons, bifidobacteria are now widely recognized as key members of the human gut microbiota, being frequently used as functional ingredients in food products. 

Some bifidobacterial species have been reported to grow and survive in milk and dairy products [22,23]. This observation is supported by the genomic dissection of species-specific metabolic capabilities across the whole genus that highlights a range of bifidobacterial species possessing genes dedicated to the utilization of carbohydrates typically found in dairy matrices [19]. Furthermore, a recent strain-level assessment of horizontal transmission of bacteria across the Parmigiano Reggiano cheese production chain revealed that a bifidobacterial species harbored by dairy cattle and their associated environment are transferred to cheese produced from their raw milk, where it may colonize and persist in the consumers’ gut [23]. These data not only highlight that bifidobacteria may modulate the cheese microbiota, thus perhaps participating in the development of the organoleptic features of cheese, but that they may also modulate the gut microbiota of human consumers.

Although these findings depict this genus as a member of the cheese microbiota exploiting milk as a vector, the distribution of bifidobacteria in fermented dairy products has never been assessed in detail. For this reason, we performed 16S rRNA gene-based microbial profiling and bifidobacterial ITS-based profiling of 21 cheeses that represent the most commonly consumed Italian cheeses made from unpasteurized milk. Notably, while profiling based on 16S rRNA, the gene is accurate only down to the genus level, but the ITS profiling approach allows an in depth taxonomic reconstruction of bifidobacterial communities down to the subspecies level. 

## 2. Methods

### 2.1. Sample Collection

All samples were kept on ice and shipped to the laboratory under frozen conditions where they were preserved at −80 °C until further processing. 

### 2.2. Bacterial DNA Extraction, 16S rRNA Gene PCR Amplification, and Sequencing

Aliquots of cheese samples collected without RNAlater were subjected to bacterial DNA extraction using the QIAamp DNA Stool Mini Kit following the manufacturer’s extraction (Qiagen, Hilden, Germany). Partial 16S rRNA gene sequences were amplified from extracted DNA using primer pair Probio_Uni/Probio_Rev. targeting the V3 region of the 16S rRNA gene sequence [24]. Illumina adapter overhang nucleotide sequences were added to the partial 16S rRNA gene-specific amplicons, which were further processed involving the 16S Metagenomic Sequencing Library Preparation Protocol (Part #15044223 Rev.—Illumina). Amplifications were carried out using a Verity Thermocycler (Applied Biosystems, Foster City, CA, USA). The integrity of the PCR amplicons was analyzed by electrophoresis on a 2200 TapeStation Instrument (Agilent Technologies, Santa Clara, CA, USA). DNA products obtained following PCR-mediated amplification of the 16S rRNA gene sequences were purified by a magnetic purification step employing the Agencourt AMPure XP DNA purification beads (Beckman Coulter Genomics GmbH, Bernried, Germany) in order to remove primer dimers. DNA concentration of the amplified sequence library was determined by a fluorometric Qubit quantification system (Life Technologies, Carlsbad, CA, USA). Amplicons were diluted to a concentration of 4 nM, and 5 µL quantities of each diluted DNA amplicon sample were mixed to prepare the pooled final library. Sequencing was performed using an Illumina MiSeq sequencer with MiSeq Reagent Kit v3 chemicals.

### 2.3. 16S rRNA/ITS Microbial Profiling

Partial 16S rRNA gene sequences were amplified from extracted DNA using primer pair Probio_Uni/Probio_Rev, targeting the V3 region of the 16S rRNA gene sequence [24]. Partial ITS sequences were amplified from extracted DNA using primer pair Probio-bif_Uni/Probio-bif_Rev, which targets the spacer region between the 16S rRNA and the 23S rRNA genes within the ribosomal RNA (rRNA) locus [11]. Illumina adapter overhang nucleotide sequences were added to the partial 16S rRNA gene-specific amplicons and to the targeted ITS amplicons of approximately 200 bp, which were further processed employing the 16S Metagenomic Sequencing Library Preparation Protocol (Part #15044223 Rev. B—Illumina). Amplifications were carried out using a Verity Thermocycler (Applied Biosystems). The integrity of the PCR amplicons was analyzed by electrophoresis on a 2200 TapeStation Instrument (Agilent Technologies). DNA products obtained following PCR-mediated amplification of the 16S rRNA gene sequences were purified by a magnetic purification step involving the Agencourt AMPure XP DNA purification beads (Beckman Coulter Genomics GmbH, Bernried, Germany) in order to remove primer dimers. DNA concentration of the amplified sequence library was determined by a fluorimetric Qubit quantification system (Life Technologies). Amplicons were diluted to a concentration of 4 nM, and 5 µL quantities of each diluted DNA amplicon sample were mixed to prepare the pooled final library. Sequencing was performed using an Illumina MiSeq sequencer with MiSeq Reagent Kit v3 chemicals.

### 2.4. 16S rRNA/ITS Microbial Profiling Analysis

Following sequencing, the fastq files were processed using QIIME2 software [25]. Paired-end reads were merged, and quality control retained sequences with a length between 140 and 400 bp, mean sequence quality score >25, and with truncation of a sequence at the first base if a low quality rolling 10 bp window was found. Sequences with mismatched forward and/or reverse primers were omitted. 16S rRNA gene and bifidobacterial ITS operational taxonomic units (OTUs) were defined at 100% sequence homology using DADA2 [26] and OTUs with less than 2 sequences in at least one sample were removed. All reads were classified to the lowest possible taxonomic rank using QIIME2 [25,27] and a reference dataset from the SILVA database [28], in case of 16S rRNA gene sequences, or a custom bifidobacterial ITS database [11]. Biodiversity of the samples (alpha-diversity) was calculated with Chao1 index. 

### 2.5. Statistical Analysis

All statistical analyses (i.e., ANOVA, PERMANOVA, Student’s t-test as well as the Kendall tau rank co-variance analysis) were performed with SPSS software v. 22 (IBM SPSS Statistics for Windows, Version 22.0. Armonk, NY, USAS: IBM Corp.). The force-driven network was created using Gephi (Available online: https://gephi.org/) and modularity was defined with a resolution of 0.6.

## 3. Results and Discussion

### 3.1. Dissecting the Distribution of Bifidobacteria Across Italian Cheese

Samples of 21 Italian cheeses were collected in order to obtain a general overview of the distribution of bifidobacteria in such fermented dairy products (Table S1). We focused on cheeses produced from raw milk (i.e., without any pasteurization step or any other treatments that may have negatively affected bacterial viability), in order to include only dairy products that are more likely to harbor living bacteria (Table S1). Due to its relevance as a commonly consumed dairy product, ricotta was also included in the sampled cheeses. It is relevant to underline that ricotta is a derivate of whey obtained from raw milk that is subsequently cooked at a temperature of >80 °C, which is likely to kill most (vegetative) bacterial cells though it will not destroy DNA and will therefore still produce a metagenomic profile. All samples were subjected to 16S rRNA gene microbial profiling for reconstruction of the taxonomic composition at the genus level, generating a total of 973,861 sequence reads, with an average of 46,374 reads per sample (Table S1).

**Figure 1:**
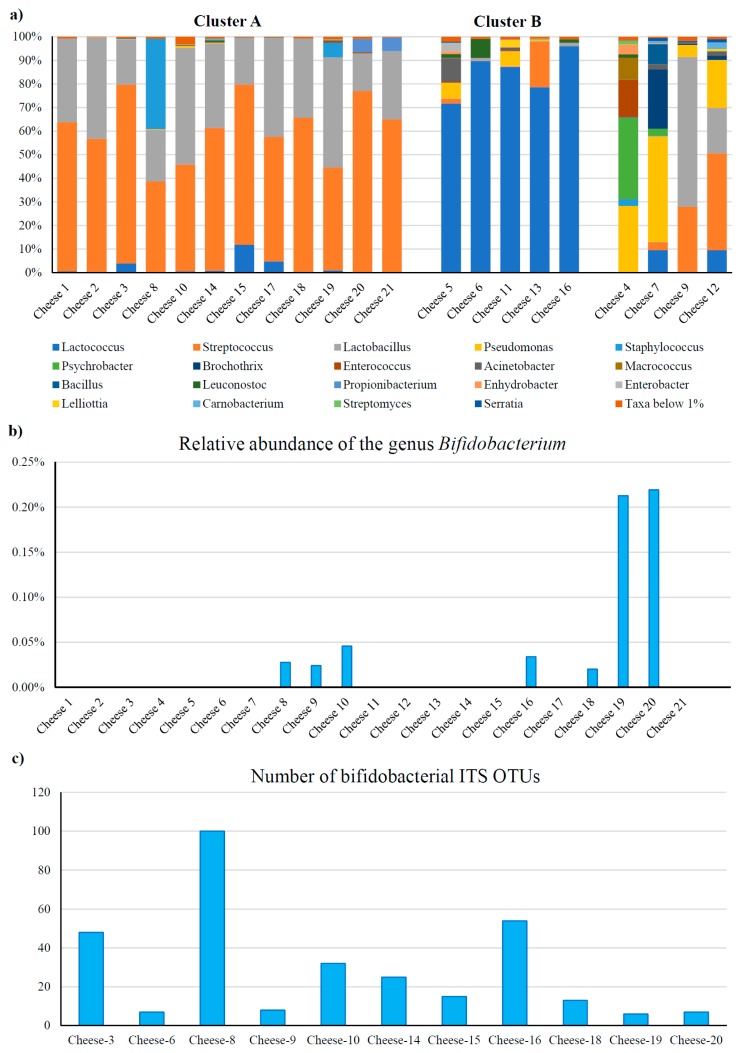
Taxonomic dissection of the raw cheese microbiota. (**a**) shows a bar plot representation of the taxonomic composition at the genus level of the profiled microbiota from cheese samples included in this study. Only taxa with relative abundance of >1% are shown. (**b**) reports the relative abundance of bifidobacteria observed by 16S rRNA gene microbial profiling data in the 21 raw cheese samples. (**c**) depicts the bifidobacterial biodiversity, reported as the number of operational taxonomic units (OTUs), obtained from bifidobacterial internally transcribed spacer (ITS) profiling data.

Sequencing data were used to generate rarefaction curves of the alpha-diversity based on the observed OTU index. The obtained graph showed that all sequenced samples tend to reach a plateau, thus indicating that our sequencing efforts covered the vast majority of the biodiversity present in the cheeses that had been included in the analysis (Figure S1). Intriguingly, the three fresh (non-aged) ricotta (cheese samples 7, 9, and 10) included in this study showed higher microbial biodiversity compared to other cheeses, including aged ricotta (cheese sample 8). This may reflect the fact that, in contrast to other cheeses included in this study, ricotta is a “whey cheese” produced from coagulation of proteins present in whey (i.e., the liquid remaining after the milk has been curdled and strained). Moreover, the cooking at >80 °C step required for ricotta production may lead to the inclusion of a small portion of dead cells in the metagenomic profiles obtained, thus leading to higher observed biodiversity. Nevertheless, as shown below, the taxonomic and beta-diversity data obtained did not reveal any major shift in the overall profiles when compared to other raw milk cheeses (Figure S2, Figure 1), thus ricotta samples were included in all further analyses.

Beta-diversity analysis based on the Bray–Curtis index calculated for genus-level profiles was also conducted and represented through PCoA (Figure S2). Interestingly, we observed two clusters, named Cluster A and Cluster B, constituted respectively by 14 and five cheeses with similar profiles (Figure S2). 

In order to elucidate the taxonomic differences between cheese constituting the two clusters that were observed by beta-diversity analysis, we analyzed the genus-level composition obtained from 16S rRNA gene microbial profiling data (Figure 1). Notably, samples establishing Cluster A were shown to be dominated (total relative abundance >90%) by the genera *Streptococcus* and *Lactobacillus*, with the exception of sample Cheese 8 showing 38.2% of *Staphylococcus* (Figure 1). Manual classification of 16S OTUs corresponding to *Staphylococcus* found in Cheese 8 resulted in its putative designation to species *Staphylococcus equorum*. Interestingly, a subspecies of this taxon was previously isolated from Swiss mountain cheeses [29]. Furthermore, Cluster B included the cheese samples whose microbiota was dominated (>70%) by *Lactococcus*, while cheese samples falling outside these clusters were shown to exhibit variable profiles (Figure 1).

Intriguingly, cheese samples showing the highest microbial biodiversity (>40 OTUs) are widespread across the whole PCoA representation (Figure S2), also falling in Clusters A and B, thus indicating the relevance of low-abundance components of the cheese microbiota in defining the overall biodiversity (Figure S2). In addition, we also observed that three of the four Toma cheeses included in this study, encompassing two with high microbial biodiversity (Cheese 4 and 5) and two with low microbial biodiversity (Cheese 6 and 13), fall in Cluster B (Figure S2), thus indicating that cheesemaking of Toma cheese favors higher variability in the microbiota biodiversity, probably due to environmental effects linked to the different production sites such as milk microbiota, while supporting high abundance of *Lactococcus* genus (Figure 1).

Together, these data indicate that *Streptococcus*/*Lactobacillus*-dominant and *Lactococcus*-dominant microbiota represent the most prevalent cheese community state types (cheese CST) in raw cheese produced with unpasteurized milk, named respectively cheese CST 1 and cheese CST 2.

In this context, it is worth mentioning that we could not find any correlation between production site, use of natural whey starters (back-slopping and no specific bacterial starters were used for the sampled cheeses) or cheese ripening (Table S1) and cheese CST. Intriguingly, this may indicate that complex environmental factors or/and specific characteristics of strains persisting in the production chain may be responsible for the establishment of the specific equilibrium among cheese microbiota members.

Assessment of the relative abundance of bifidobacteria across the sampled cheeses revealed that the *Bifidobacterium* genus could be detected only in seven samples with a relative abundance ranging from 0.02% to 0.22% (Figure 1). Notably, this indicates that bifidobacteria represent just a minor microbial component in raw cheese and that their abundance is probably linked to environmental peculiarities of each production site.

Using the seven samples with detectable levels of the genus *Bifidobacterium*, we performed an evaluation of co-variances between bifidobacteria and other members of the cheese microbiota based on the Kendal index. Notably such analyses revealed that the presence of bifidobacteria positively correlates with the presence of the genus *Propionibacterium* (co-variance of 0.845, *p*-value < 0.05).

In the context of this study, we also evaluated if the use of starter cultures/natural whey or thermal treatments at a temperature of >50 °C during the production of the sampled cheeses may impact their microbiota composition. Notably, we did not identify any clear correlation. Nevertheless, a higher sampling size is needed for a statistically robust assessment of these correlations in order to determine the precise impact of geographical localization/production site in defining the microbial composition.

### 3.2. Assessment of The Bifidobacterial Population at The Subspecies Level

The availability of a reliable tool for subspecies classification of members of the genus *Bifidobacterium*, which is based on sequencing of the internally transcribed spacer (ITS) sequence [11], allowed us to perform a precise profiling of the bifidobacterial communities across the sampled cheeses. Notably, since this approach relies on targeted amplification of the ITS sequence of bifidobacteria through genus-specific primers, we were able to reconstruct the bifidobacterial population of 12 cheeses encompassing five samples for which the presence of this genus could initially not be detected by 16S rRNA gene-based sequencing data (Table S1). The latter was probably caused by the low relative abundance of bifidobacterial when compared to the total microbial population. Sequencing of the ITS amplicon produced a total of 66,011 reads, with an average of 5501 reads per sample (Table S1). Due to the low number of reads obtained for sample Cheese 5 (245), it was excluded from further analyses.

Alpha-diversity analysis based on the number of observed OTUs showed that the biodiversity of the bifidobacterial population may vary considerably, ranging from six to 100 OTUs (Figure 1), disregarding sequencing depth and cheese type, thus emphasizing the relevance of environmental effects associated with each production site in defining the cheese microbiota.

**Figure 2:**
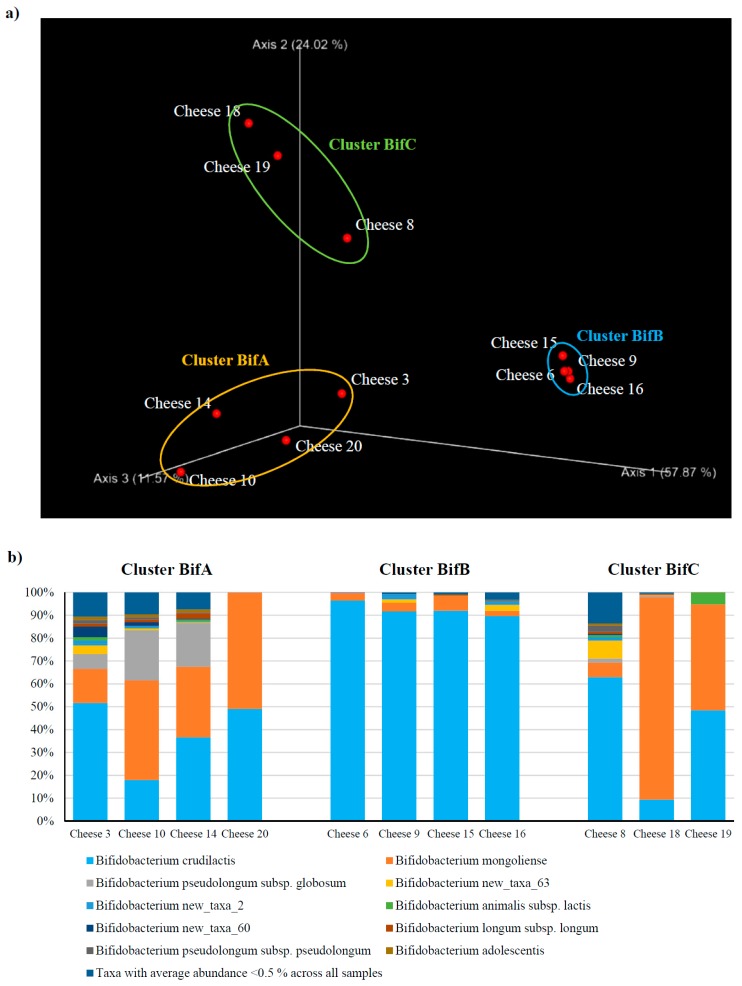
Taxonomic dissection of the bifidobacterial population harbored by raw cheese. (**a**) shows a PCoA representation of the beta-diversity analysis performed for bifidobacterial ITS data at species level using the Bray–Curtis index. (**b**) displays a bar plot representation of the bifidobacterial population at the species level observed in 11 ‘raw’ cheese samples (i.e., those for which we could obtain data). Only taxa with an average abundance >0.5% are shown.

Moreover, beta-diversity analysis obtained using the Bray–Curtis index and species-level profiles generated a PCoA representation that allowed identification of three clusters named BifA, BifB, and BifC (Figure 2). Inspection of the taxonomic profiles at species level revealed that the microbiota of cheese included in clusters BifA and BifC are dominated by both *Bifidobacterium mongoliense* and *Bifidobacterium crudilactis*, with cluster BifA showing a higher abundance of *Bifidobacterium pseudolongum* subsp. *globosum* (average of 11.7%) (Figure 2). In contrast, cluster BifB is characterized by a dominance of *B. crudilactis* (average of 92.9%) with much lower average abundance of *B. mongoliense* (average of 4.0%) (Figure 2). Notably, these findings confirm previous observations about the adaptation of *B. crudilactis* and *B. mongoliense* to grow and survive in milk and cheese environments [22,23,30,31], and reveal the widespread distribution of these bifidobacterial species in cheeses produced from raw milk derived from cow, buffalo, sheep, and goat.

Further analysis of the taxonomic profiles also showed that seven additional species are present with an average relative abundance of >0.5% of the whole bifidobacterial population (Figure 2). These taxa encompass four known species (i.e., *Bifidobacterium animalis* subsp. *lactis*, *Bifidobacteirum longum* subsp. *longum*, *Bifidobacterium pseudolongum* subsp. *pseudolongum*, and *Bifidobacterium adolescentis*), along with three putative novel bifidobacterial species named new_taxa_2, new_taxa_60, and new_taxa_63 based on previously defined nomenclature [12] (Figure 2). Interestingly, isolation and genomic characterization of these putative novel taxa will be relevant for a complete understanding of the bifidobacterial ecology in cheese environments.

### 3.3. Co-Variances between Bifidobacteria and Other Cheese Colonizers

**Figure 3:**
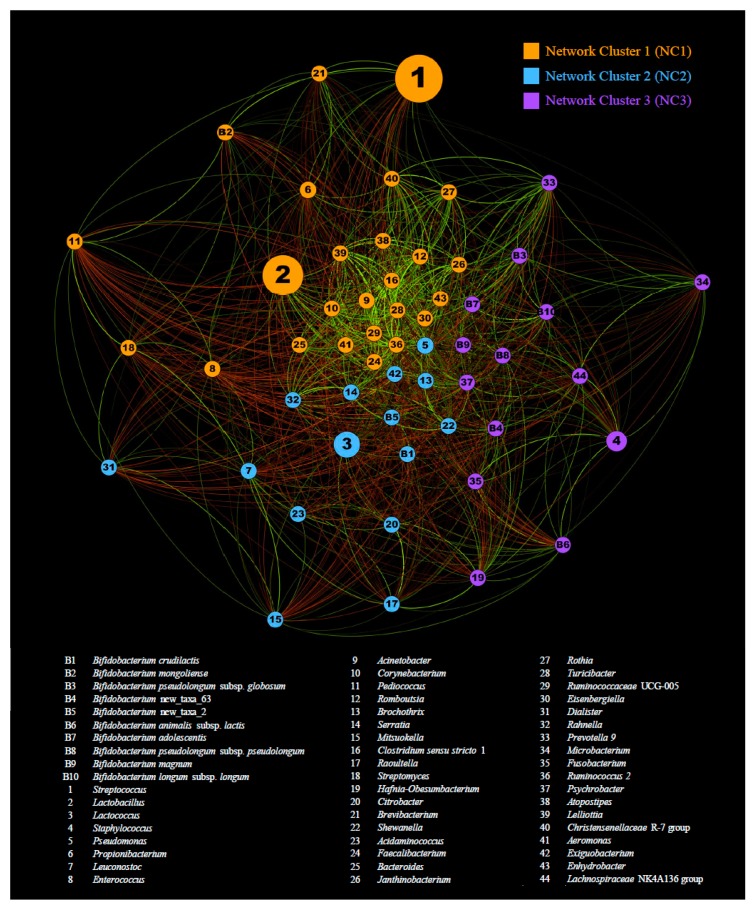
Force-driven network representation of co-variances among bifidobacterial species and other genera in raw cheese. The force-driven network was generated using taxa as nodes and co-variances as edges. Only genera with a relative abundance of >0.01% and bifidobacterial species with normalized relative abundance > 0.0001% were included in the analysis. Edge color indicates positive correlations (in green) and negative correlations (in red).

In order to evaluate how the bifidobacterial population modulates and is modulated by non-bifidobacterial components of the cheese microbiota, we performed normalization of ITS profiles with relative abundance of bifidobacteria detected by 16S rRNA gene microbial profiling. Profiling data of the 10 bifidobacterial species showing an average abundance of >0.0001% after normalization, when compared to the total bacterial population, were used to evaluate co-variances with genera showing an average relative abundance of >0.01%. The resulting covariance matrix was then used to compute a force-driven network (Figure 3). Intriguingly, modularity assessment performed through Gephi software revealed the presence of three main clusters of covariant taxa. Remarkably, the network cluster NC1 covers the genera *Streptococcus* and *Lactobacillus* (Figure 3) (i.e., the dominant taxa observed in cheese CST 1), while the cluster NC2 covers the genus *Lactococcus* (Figure 3), the dominant taxa found in cheese CST 2. Moreover, the third cluster predicted by the modularity analysis encompasses the fourth most abundant genera observed in raw cheese (i.e., *Staphylococcus*) (Figure 3). Intriguingly, these data reveal that the dominant genera found in raw cheese modulate the whole microbiota by covariance with a range of accessory low-abundance taxa. Focusing on the most abundant species encompassing the bifidobacterial population of raw cheese (see above), *B. mongoliense*, *B. crudilactis*, and *B. pseudolongum* subps. *globosum* cluster in NC1, NC2, and NC3, respectively. 

Remarkably, these findings highlight that the species-level composition of accessory genera present at low relative abundance, such as the bifidobacterial population, are shaped by an intricate network of covariances with the dominant genera harbored by raw cheese.

## 4. Conclusions

The ecology of microbial populations constituting the cheese microbiota made from raw milk has never been assessed in detail using metagenomic-based approaches. For this reason, 16S rRNA gene-based microbial profiling was employed to obtain a comprehensive view of the genus-level composition, while bifidobacterial ITS profiling allowed an in-depth exploration of the taxonomic composition of the *Bifidobacterium* genus at the species level. The obtained results revealed community state types typical of the ‘raw’ cheese microbiota, named cheese CST1 and cheese CST2, characterized by dominance of *Streptococcus*/*Lactobacillus* or *Lactococcus* genera. In addition, data collected suggest that the genus *Staphylococcus* also plays a dominant role in a limited number of cases, thus additional screening of raw cheese samples is needed to confirm this assumption. Furthermore, in-depth analysis of the ITS profiling data showed that *B. mongoliense* and *B. crudilactis* constitute the dominant members of the bifidobacterial population and define specific bifidobacterial community state types, thereby confirming previous observations that their genetic repertoire supports colonization of the ecological niches of milk and cheese.

Intriguingly, covariance analyses encompassing 16S rRNA and ITS-based taxonomic profiles revealed that the four dominant genera found in raw cheese (i.e., *Streptococcus*, *Lactobacillus*, *Lactococcus*, and *Staphylococcus*), shape the whole raw cheese microbiota through a complex network of covariances that modulate even low relative abundance genera with species-level resolution.

Data collected in this study underline that complete understanding of the intricate relationships between members of the microbiota of cheese produced using unpasteurized milk will be of key industrial relevance due to the crucial role exerted by these bacteria in defining the organoleptic features of corresponding fresh and aged cheese products. 

## Data Availability

Raw sequences of 16S rRNA gene profiling data coupled with ITS profiling data are accessible through SRA study accession number PRJNA574054.

## Supplementary Materials

The following are available online at https://www.mdpi.com/2076-2607/7/12/599/s1, Table S1. Quality filtering table of cheese included in this study. Figure S1. Evaluation of alpha-diversity of the raw cheese microbiota. Panel a shows rarefaction curves of the number of observed 16S rRNA gene OTUs generated at 100 % identity that were obtained for the 21 ‘raw’ cheese samples included in this study. Panel b illustrates through a bar plot the number of observed 16S rRNA gene OTUs generated at 100 % observed at 20,000 reads. Figure S2. Beta-diversity analysis of the raw cheese microbiota. The PCoA representation was obtained from inter-variability assessment through the Bray–Curtis index and genus-level profiles retrieved from 16S rRNA gene microbial profiling data.
