# Supplementary figures and images for: Bifidobacterial Distribution Across Italian Cheeses Produced from Raw Milk
Source: Microorganisms. 2019 Nov 21;7(12):599. doi: 10.3390/microorganisms7120599 (PMC6955966; doi:10.3390/microorganisms7120599)

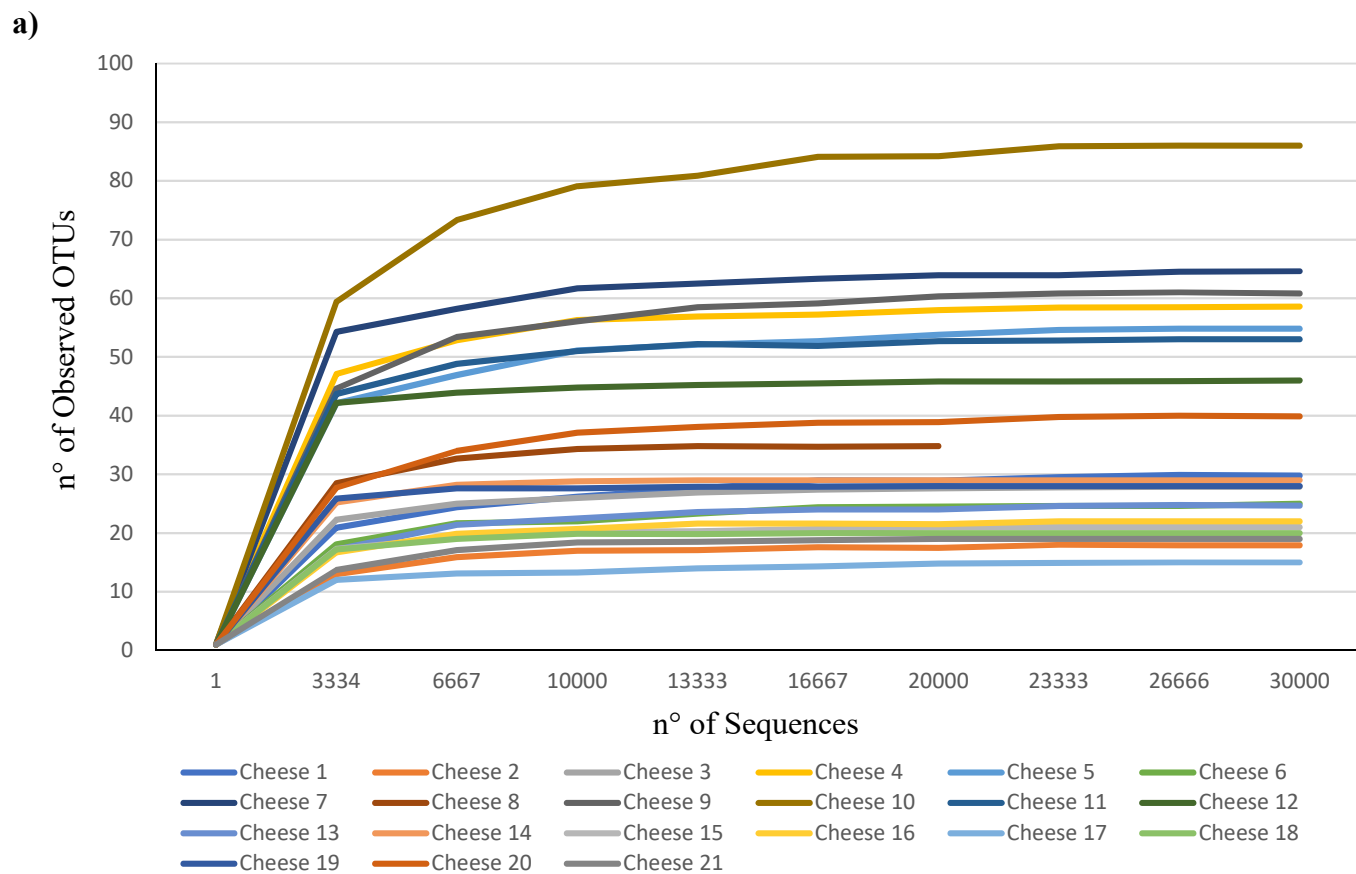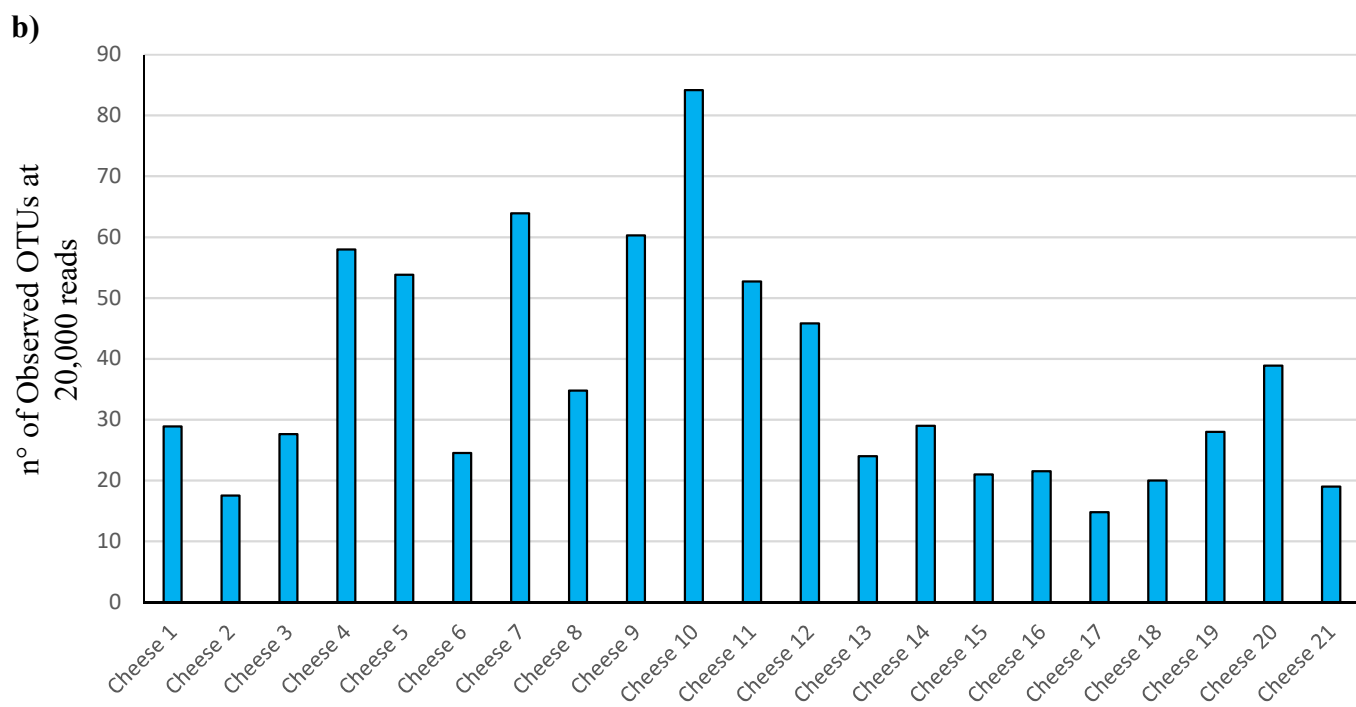

**Figure S1**

Supplement: Supplementary file 1 [file microorganisms-07-00599-s001.zip › Figure_S1.pdf]

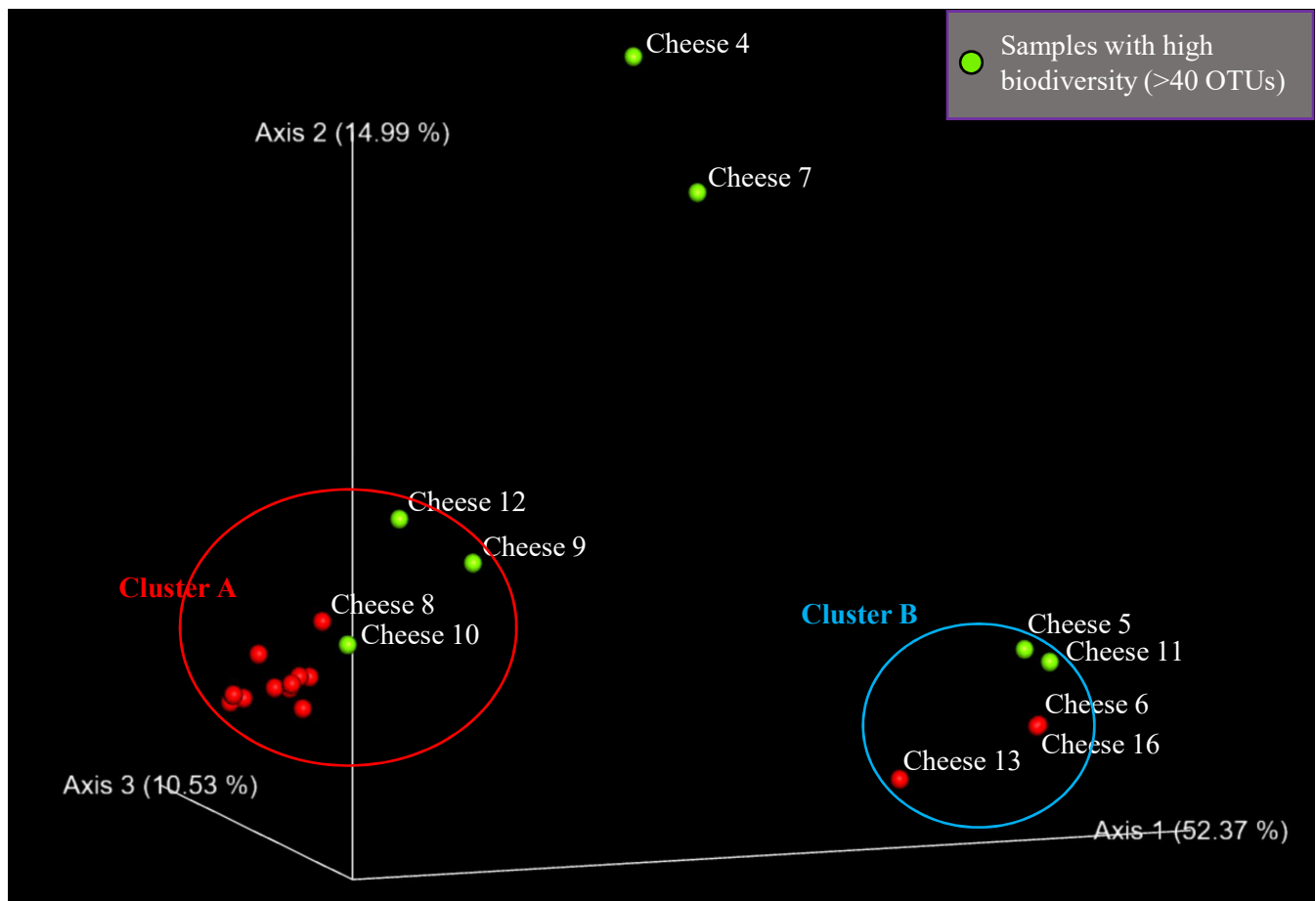

Figure S2

Supplement: Supplementary file 1 [file microorganisms-07-00599-s001.zip › Figure_S2.pdf]
